# Effects of ageing and Alzheimer disease on haemodynamic response function: a challenge for event-related fMRI

**DOI:** 10.1049/htl.2017.0005

**Published:** 2017-06-26

**Authors:** Davud Asemani, Hassan Morsheddost, Mahsa Alizadeh Shalchy

**Affiliations:** 1 Division of Radiology, Medical University of South Carolina, Charleston, SC 29407, USA; 2 Biomedical Engineering Department, K. N. Toosi University of Technology, Tehran, Iran

**Keywords:** diseases, haemodynamics, biomedical MRI, neurophysiology, deconvolution, geriatrics, signal denoising, medical signal processing, ageing, Alzheimer disease, haemodynamic response function, event-related fMRI, functional magnetic resonance imaging, brain images, neuronal activity, HRF, blood-oxygenation, deconvolution

## Abstract

Functional magnetic resonance imaging (fMRI) can generate brain images that show neuronal activity due to sensory, cognitive or motor tasks. Haemodynamic response function (HRF) may be considered as a biomarker to discriminate the Alzheimer disease (AD) from healthy ageing. As blood-oxygenation-level-dependent fMRI signal is much weak and noisy, particularly for the elderly subjects, a robust method is necessary for HRF estimation to efficiently differentiate the AD. After applying minimum description length wavelet as an extra denoising step, deconvolution algorithm is here employed for HRF estimation, substituting the averaging method used in the previous works. The HRF amplitude peaks are compared for three groups HRF of young, non-demented and demented elderly groups for both vision and motor regions. Prior works often reported significant differences in the HRF peak amplitude between the young and the elderly. The authors’ experimentations show that the HRF peaks are not significantly different comparing the young adults with the elderly (either demented or non-demented). It is here demonstrated that the contradictory findings of the previous studies on the HRF peaks for the elderly compared with the young are originated from the noise contribution in fMRI data.

## 1 Introduction

The Alzheimer disease (AD) is an advanced neurodegenerative illness. AD generally causes a decline in cognitive tasks such as progressive loss of memory, reasoning and language. This disease mostly happens in the patients of the ages between 40s and 90s [1] such that age is considered as a major risk factor. In developed countries, the incidence rate is 8 and 30% for the individuals over 65 and 85 years, respectively. AD often progresses gradually and the patients can seldom survive more than 8–10 years after the beginning of symptoms [2].

Current consensus statements have emphasised the need for the early diagnosis of AD [3]. However, there is actually no single and comprehensive method for diagnosis of AD [4]. A well-trained physician with an expertise in dementia may diagnose AD based on medical history, physical examination, cognitive assessment tests and laboratory and brain imaging results [4]. The diagnosis is currently made using the criteria provided with by National Institute of Neurological and Communicative Disorders and Stroke – Alzheimer's disease and Related Disorders Association [4]. Typically, it takes a few weeks to complete a diagnostic evaluation of the AD [3].

As mentioned, the detailed diagnosis of AD is primarily based on clinical, neuropsychological and imaging evaluation. The choice for the modality of the brain imaging [computed tomography (CT), magnetic resonance imaging (MRI), single photon emission CT (SPECT), positron emission tomography (PET) and functional MRI (fMRI)] depends on the duration and severity of the condition. Since MRI or CT scans cannot show brain tissue loss affecting the brain activity, SPECT, PET or fMRI is often exploited to detect the abnormalities for an early diagnosis [5, 6].

fMRI is advantageous compared with PET modality owing to its higher spatial and temporal resolution. In fMRI, the brain activity may be explored during cognitive, sensory and motor task. Hence, it can be supposed as powerful means to identify disrupted neuronal circuits underlying disorders such as AD. Considering the cortices affected by AD, a ‘cognitive stress test’ can be used to bring out subtle brain abnormalities that would otherwise remain undetected during a resting state [7].

During fMRI procedure, a brief focal neural activation evokes what is called a haemodynamic response function (HRF). The physiological processes underlying the blood-oxygenation-level-dependent (BOLD) signal and HRF, including neural metabolism, extracellular signalling, and cerebrovascular response are known to vary with age [8–11]. The BOLD signal is modelled as the product of convolution between this time-invariant HRF and an impulse train of neuronal events [12]. However, the effects of ageing on fMRI have been still undiscovered.

Several prior studies comparing haemodynamic response properties in the young and non-demented and demented elderly adults using fMRI data have led to partially-opposite results [13–19] (summarised in Table 3). In [15], Aizenstein *et al.* investigated haemodynamic responses of the young and non-demented elderly adults using an event-related (ER) sensory–motor paradigm. In their study, the HRF has been simply extracted by averaging procedure. They found no significant difference for the HRF peaks between the young and non-demented elderly adults in either vision or motor region-of-interests (ROIs). In [16], Huettel *et al.* and [18] D'Esposito *et al.* reported similar result using averaging method as well, though implicitly reported an evident decrease of signal-to-noise ratio (SNR) for the elderly.

In contrast to these results, in [14] Mohtasib *et al.* reported, though cerebral blood flow response remains at the same level, but the BOLD response significantly intensifies for the old subjects in comparison with the young group. Then, the increase of BOLD peak with ageing was interpreted to be associated with a significant reduction in either the oxygen metabolism response or the neural activity. Using an ER sensory–motor paradigm and averaging scheme, in [17] Buckner *et al.* compared HRFs for the young and non-demented and demented elderly adults as well. According to their study, HRF peaks appear to be significantly different between the young and the non-demented and demented elderly adults in vision ROI. The motor ROI was nevertheless reported to exhibit no significant difference in the HRF peaks, but with a subtle time shift for the young compared with the old. Considering a block design paradigm, in [19] Ross *et al.* reported a significant decrease in the HRF peak for the non-demented elderly adults compared with the young in vision ROI. On the opposite, in [13] Gauthier *et al.* also found a decrease in the HRF peak for the non-demented elderly adults in comparison with the young, though neuronal activity and haemodynamic response were assumed to remain unchanged through the lifespan.

Trying to interpret the inconsistency of the results associated with prior works, in [15] Aizenstein *et al.* suppose the abnormality to appear because of the negative deactivating voxels included in the HRF estimation. They address the problem of the reduced activity or HRF peak, already reported for the elderly, to the inclusion of these voxels in HRF estimation. In this paper, the contradictory results are analysed and it is demonstrated that the inconsistent findings have been originated from the simple averaging algorithm shared by all the previous works in the HRF estimation. Averaging method exhibits a large sensitivity to outliers. Also, the estimation worsens when the number of samples is low so that the background resting-state signal and noise terms appear to be non-stationary. In fMRI case, there are only a very small number of samples either [here, four samples at each time of repetition (TR) period]. Moreover, it is should be reminded that the noise contribution in fMRI data is much more critical for the elderly subjects [16, 18, 20, 21].

It is still a research challenge. Moreover, there are contradictory findings reported in the literature. This work tries to deal with this challenge and to show some probable reasons for contradictory findings of earlier works. In this paper, it is proposed to use a different procedure for extracting HRF to avoid the above-mentioned problems. BOLD time series are here modelled as the product of convolution operation between an invariant HRF and an impulse train of stimuli [12]. Then, the averaging is then replaced with deconvolution method for estimating HRF. Extracting HRF using deconvolution method has also been used in other previous works [22–28]. Selection of active voxels is here realised by statistical *t*-test (only positive *t*-values) as mentioned in Aizenstein *et al.* [15].

Due to high intrinsic noise in the functional MR images and the weakness of the BOLD signal, the fMRI images suffer from low SNR [29, 30]. The SNR becomes much lower for the ER design experiments [31] where the subject receives a short stimulus or performs single instance tasks in response to intermittently-presented stimuli. Consequently, fMRI images are required to be preprocessed before any statistical analysis. Furthermore, denoising is a common preprocessing step that is applied before analysing the fMRI data. To more suppress the noise effects, minimum description length (MDL)-based wavelet method is here utilised as an extra denoising step before HRF extraction [32–36].

## 2 Materials and methods

### 2.1 Individuals

Forty-one right-handed native English speakers took part by being paid $75. Fourteen young subjects consisting of five males between 18 and 24 years (mean 21.1 years) and without any history of neurological or visual illness. Twenty-seven older subjects were picked up out of the Washington University Alzheimer's Disease Research Center (ADRC) registry. The non-demented and demented were 14 (five males, 66–89 years with mean age of 74.9 years) and 13 subjects (six males, 68–83 years with mean age of 77.2 years), respectively. All the elderly had normal visual legerity (corrected) and checked out regarding the neurologic, psychiatric or medical illnesses being associated with dementia.

All the subjects either non-demented (control) or with demented subjects had an exam regarding the clinical dementia rating (CDR). Using CDR, the subjects with normal or mild dementia of the Alzheimer's type (DAT) were clinically categorised into: CDR0: no dementia; CDR0.5 and CDR1.0 relating to very mild and mild DAT, respectively [37]. This method has shown an accuracy about 93% for diagnosing the DAT even at the early stages (CDR 0.5) confirmed by the neuropathology validation [38]. The demented subjects in this study included 13 patients, 8 individuals showed up with very mild dementia (CDR 0.5) and the rest (5 individuals) with mild DAT (i.e. CDR 1). The control non-demented subjects ranked with CDR 0.

### 2.2 Stimuli

A Power Macintosh computer (Apple, Cupernico, CA) was used to control the stimulus display (PsyScope software) [39]. A PsyScope button box was connected to a fibre-optic light-sensitive push-button key for recording the key press response (Carnegie Mellon University, Pittsburgh, PA). The buttons are all covered to avoid any response intricacy except one. A screen was used to project the stimuli (Am- Pro Model LCD-150, Ampro, Melbourne, FL) and was placed at the back of the magnet bore. The subjects were able to see the display through a mirror that was attached to the head coil. As the most of the elderly subjects were needing corrective lenses to see, they were provided with magnetic-compatible glasses.

The task paradigm was basically composed as follows: (i) a visual stimulus was presented for 1.5 s; (ii) each subject then presses a key with his right index as soon as the stimulus begins. The visual stimulus included a checkerboard of 8 Hz counter phase flickering (black to white) that was subtending about 128 of visual angle (68 in each visual field) as used by Miezin *et al.* [40].

The trials were carried out in two distinct conditions. The stimuli were presented either each one in isolation, or in pair come apart with 5.36 s as inter-trial interval. Every trial condition lasted for 21.44 s to accomplish an eight-image acquisition. Every subject performed 60 trials, in 4 runs, i.e. each run included 15 trials. Those two conditions were pseudo-randomly distributed and intermixed. Then, the evoked haemodynamic response could be obtained for an isolated and transient event [41].

### 2.3 Image acquisition

The MRI scans were acquired using a 1.5 T Vision System (Erlangen, Germany) system with a head coil being standard circularly polarised. The scan parameters were: 128 images with TR = 2.68 s, TE = 30 ms, 90° flip angle, 30 slices (8 mm slice thickness), and the slice plane resolution of 3.75 × 3.75 mm^2^. Each run collected 128 total images of sequential whole brain in four trials for each subject. Total four runs lasted 5.5 min with a 2 min delay between runs for each subject. The subjects got a rest during delay intervals.

### 2.4 fMRI data preprocessing

To make sure about longitudinal magnetisation stabilisation, four initial volume images were discarded. Then, preprocessing steps were applied including correction of even/odd slice timing and realignment or motion correction (SPM software) [42]. Next, the vision and motor cortices were selected as desired ROI. Visual cortex included the occipital cortex, the cuneus and the precuneus. Also, the motor cortex was refined to the precentral gyrus and the supplementary motor area. The AAL map was employed from MRIcro software package [43]. Next, the selected vision and motor cortices were applied to MNI single-subject's space as well.

The anatomical and functional images were coregistered using the standard MNI space for each subject in two-step registration as follows. First, mean functional image was obtained and aligned with the respective anatomical image. Then, the anatomical image was registered to the MNI template. Lastly, the related transformation matrices were concatenated. That concatenated transformation matrix were then applied to all the functional scans for registering to MNI standard template. The alignment was realised with 12 DOF as affine transformation in FSL package [44]. Finally, the mask was applied to the images for extracting the functional sequences associated with visual and motor cortices [45].

A visual check was also carried out for all the subjects to make sure about the accuracy of ROI selection.

### 2.5 fMRI data analysis

#### 2.5.1 ROI selection

The time series of visual and motor cortices were obtained from the preceding subsection. A *t*-test analysis was performed on each subject's data. According to the neuronal activation mechanism, it is supposed that the HRF peak occurs around 4–6 s after the stimulus (finger tap), here at the third scan. Subtracting the first scan from the third one, the difference is used for activation detection. To maintain the compatibility with the previous work of Aizenstein *et al.* [15] for comparison purposes, top-32 voxels have been selected with the largest positive *t*-values for studying HRF at each ROI. For ROI selection, the threshold *t*-value was independently determined for each individual subject. It should be reminded that the *t*-values of the remaining voxels have been negligible compared with the selected top-32 voxels according to the experimentations (i.e. the group of selected 32 voxels are significantly important). The activation detection was performed for both time series: original data and MDL-denoised data. According to the experimentations, a considerable part of voxels [80% of top-32 voxels (Table 1)] has changed after applying MDL denoising procedure.

**Table 1:** Per cent of voxels change

Different activated voxels in two states: without and with MDL denoising (R-MDL)	Region	
	Vision, %	Motor, %
demented adults	82.00	82.10
non-demented adults	87.17	83.22
young adults	82.93	81.68

#### 2.5.2. HRF extraction with averaging

For comparison purposes, HRF was estimated using averaging procedure like prior works as well. For each subject, the top-32 voxels were selected for both vision and motor regions. The HRF was supposed as the mean MR signal for each voxel at each eight time points (i.e. scans). Then, the average of these mean MR signals was used as the HRF. The resulting HRF was considered with respect to baseline (scan 1) in per cent for standard presentation.

#### 2.5.3. HRF extraction with deconvolution

BOLD time-series data are here modelled as the product of convolution operation between an invariant HRF and an impulse train of neural events [12]. For each subject and for both regions, top-32 voxels were considered. To estimate the HRF, the BOLD time series was deconvolved with the stimuli (train of impulses at the known onsets of the events – activity-inducing signal time) supposing a constant length for HRF. Deconvlolution algorithm has been realised in five steps [46] as follows:

- Suppose the matrix ***X*** with the size of time series × length of HRF, filled initially out with zero.
- Change ***X*** so that a vector of unity with the length of HRF is placed in each row at the onset of stimuli.
- Add a column of unity at the ninth column of ***X***, to account for estimating the baseline as a regressor to estimate the baseline.
- Estimate the HRF by calculating Moore–Penrose pseudo-inverse of matrix ***X*** and multiply the product with the BOLD time series.
- HRF is corrected with adding the baseline.

#### 2.5.4. MDL wavelet denoising

As mentioned in the previous section, it is necessary to use an extra denoising with robust performance to sufficiently suppress the noise contributions because of low SNR of BOLD signal, particularly in the elderly [29, 30]. This low SNR particularly becomes a problem for ER design experiments [31] where the subject receives a short stimulus or performs single tasks in response to intermittently presented stimuli. The noise contribution gets larger and more important as age increases [16, 18, 20, 21]. In the HRF estimation, the noise terms are much more critical. Then, an extra denoising procedure with robust performance is essential. One of the most common tools for denoising is the wavelet transform along with thresholding procedure [47, 48]. The threshold value critically determines the performance of denoising algorithms based on discrete wavelet transform (DWT) [48]. Many algorithms have been proposed for selecting this critical threshold such as VisuShrink [48], SureShrink [49], MDL or crude MDL (C-MDL) [36] and refined MDL (R-MDL) [34].

In MDL method, a cost function is proposed so that the optimum threshold may be obtained by optimising this cost function. Thus, the complexity of thresholding may be automatically controlled. In this study, the denoising is applied to fMRI applications for extracting the HRF at the activated voxels. The denoising method and particularly the thresholding step plays an important role in determining the overall performance [35].

#### 2.5.5. R-MDL algorithm

The observed time series, *y^n^* = (*y*^1^,…,*y^n^*)^T^ is assumed to have an additive noise in general linear model (GLM), as follows:

(1) $$y^n = x^n + \varepsilon ^n.\eqno\lpar 1\rpar $$

where the noise term *ɛ^n^* is often assumed to be Gaussian and *x^n^* is the desired noiseless time series (e.g. BOLD signal). In fact, the main objective is to optimally extract the uncorrupted original signal *x^n^*. In GLM method, the original signal is supposed to be the activation coefficients multiplied by the design matrix. Since, it is here desired to denoise the fMRI signal before the HRF extraction using deconvolution operation, then, the design matrix has been developed and considered in accordance with the input impulse train (stimuli vector). GLM method generally invokes the convolution operation in the matrix form (design matrix). Given the orthonormal regression matrix ***W***, the DWT of the noisy data is defined as

(2) $$c^n = {\bi W}^{\rm T}y^n.\eqno\lpar 2\rpar $$

Invoking MDL principle, the DWT coefficients may be considered as two categories: signal and noise. The MDL classification tries to achieve the minimum description of both data and the model itself simultaneously [50–53]. The length of the description is defined by the negative logarithm of the so-called normalised maximum-likelihood (NML) expression [34, 36]. The NML represents a universal model supposing a parametric distribution profile with parameters $\hat \theta \left({y^n} \right)$ as follows [54–56]:

(3) $$f_{nml}\left({y^n} \right)= \displaystyle{{\,f\left({y^n\semicolon \; \hat \theta \left({y^n} \right)} \right)} \over {\int_A {\,f\left({z^n\semicolon \; \hat \theta \left({y^n} \right)} \right)\; {\rm d}z^n} }}.\eqno\lpar 3\rpar $$

where *A* represents the set of coefficients with length *n*. $\hat \theta \left({y^n} \right)$ is the NML estimate of the parameters. The NML is used to optimise the parameters for the minimum cost.

For MDL-based denoising, the wavelet coefficients are categorised into two groups of *k* and *n* − *k* members. First group of coefficients are considered for reconstruction and the remaining *n* − *k* are set to zero. In C-MDL, a subset *γ* including the *k* largest wavelet coefficients are selected to minimise the following cost function [36]

(4) $$\displaystyle{{n - k} \over 2}\ln \displaystyle{{S\left({y^n} \right)- S_\gamma \left({y^n} \right)} \over {n - k}} + \displaystyle{k \over 2}\ln \displaystyle{{S_\gamma \left({y^n} \right)} \over k} + \displaystyle{1 \over 2}\ln k\left({n - k} \right).\eqno\lpar 4\rpar $$

Here, *S*(*y^n^*) and *S_γ_*(*y^n^*) stand for the sum squares of all wavelet coefficients and the coefficients belonging to *γ*, respectively. The number of coefficients in *γ* is equal to *k* and *n* is the length of time series. The upper limit for *k* always meets *k* < 0.95*n* [33].

In R-MDL as discussed in [34], the cost function is modified as follows:

(5) $$\displaystyle{{n - k} \over 2}\ln \displaystyle{{S\left({y^n} \right)- S_\gamma \left({y^n} \right)} \over {{\left({n - k} \right)}^3}} + \displaystyle{k \over 2}\ln \displaystyle{{S_\gamma \left({y^n} \right)} \over {k^3}}.\eqno\lpar 5\rpar $$

Larger the number of model classes, more important generally is the selection of class index. For MDL denoising, there are 2*^n^* possible conditions to select. The encoding of the class index affects so much on the performance of fMRI data denoising [34].

## 3 Results

### 3.1 Denoising effects on activation detection

**Fig. 1:**
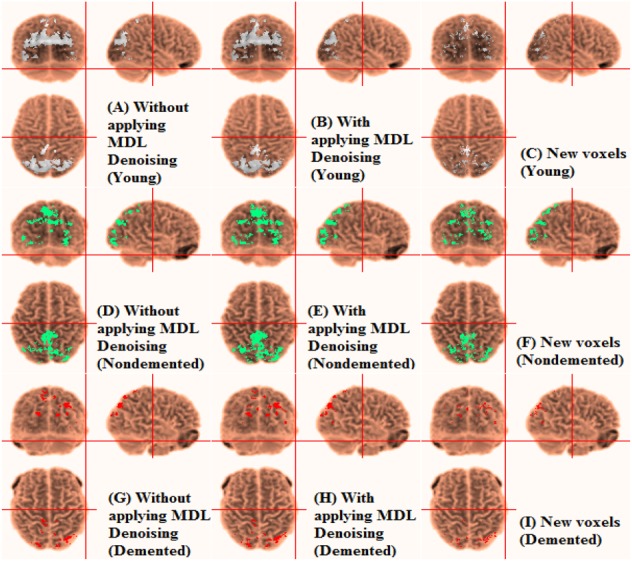
Automatic labelling of grey matter in vision. Activation detection of fMRI data in young subject *a* Without applying MDL denoising *b* With applying MDL denoising by R-MDL *c* New voxels *d* Activation detection of fMRI data in non-demented elderly adults without applying MDL denoising *e* Activation detection of fMRI data in non-demented elderly adults with applying MDL denoising by R-MDL *f* New voxels *g* Activation detection of fMRI data in demented elderly adults without applying MDL denoising *h* Activation detection of fMRI data in demented elderly adults with applying MDL denoising by R-MDL *i* New voxels

The effects of denoising fMRI data are here studied on the selection of activated voxels. The noise contribution gets larger and more important through ageing [16, 18, 20, 21]. For both the vision and motor regions, all-time series were first denoised by R-MDL for the young and non-demented and demented elderly adults as in [32, 33], followed by the *t*-test for selecting activated voxels. The activated voxels have been selected as shown in Fig. 1 (vision).

In both vision and motor regions, the initial connected regions of activated voxels (without applying MDL denoising) have been extended for both the non-demented elderly adults and the young cases after applying MDL denoising. In the non-demented elderly, new connected sub-regions or spots have nonetheless appeared as well. It may be referred to the lower SNR of fMRI data in the non-demented elderly adults (0.35) compared with the young (0.52) [16, 21]. However, for the demented elderly adults, these changes are very small. The correlation of fMRI data of different voxels may decrease because of independent noise elements. Hence, MDL denoising leads to an extension of activated voxels sub-regions. In the non-demented elderly adults, the large contribution of noise may have resulted in the disappearance of activated spots or little sub-regions.

To better evaluate the MDL denoising process, the activated voxels associated with the top32 correlation values are compared without and with applying MDL denoising. Table 1 demonstrates the per cent of activated voxels of top32 correlation being not common between two sates: without and with MDL denoising. Accordingly, the noise appears to be dominant in the activation detection, i.e. the noise component leads to different selection of voxels in the activation detection.

Table 2 demonstrates the per cent of new voxels included in the top32 activated voxels selected after applying MDL denoising to original fMRI images. Accordingly, the noise appears to be dominant in the activation detection. It is reminded that the activation detection is because the selected activated voxels will be then used for HRF estimation to contrast the non-demented and demented elderly from the young.

**Table 2:** Per cent of new voxels

New voxels in Top32 actives voxels after MDL denoising (R-MDL)	Region	
	Vision, %	Motor, %
demented subjects	48.17	46.68
non-demented subjects	43.16	57.61
young subjects	38.68	34.56

### 3.2 HRF extraction

The HRF is here estimated from the fMRI volume images. To better study the noise effects, the estimation has been here applied to the fMRI volume images before and after applying MDL denoising.

#### 3.2.1. Without MDL denoising

All previous studies [13–19] tried to investigate the changes in HRF amplitude peaks as a marker for ageing study. Also modulation of the amplitude of the HRF arising from a brief, temporally well-characterised stimulus would be consistent with neuronal mechanisms, e.g. altered firing rates and synaptic input [57]. Though, variation of the HRF shape might be associated with vascular mechanisms, e.g. vascular time constants assuming that the temporal profile of neural responses is similar across volunteers [58]. However, the neuronal mechanisms would implicitly cause vascular mechanisms (O_2_ demand etc.). Then, it may be imagined that the HRF peaks can indirectly demonstrate the vascular time constants. In the averaging method, the results show no significant difference in HRF peaks between the young and the elderly adults in both vision and motor regions (*p* > 0.1).

Considering the fMRI data without MDL denoising, in contrast with averaging method, the HRF amplitude peaks appear to be significantly different in vision region between the young and the elderly adults (*p* < 0.05). In vision cortex, the HRF peaks are significantly larger for the young compared with the elderly adults (non-demented and demented).

#### 3.2.2. With MDL denoising

**Fig. 2:**
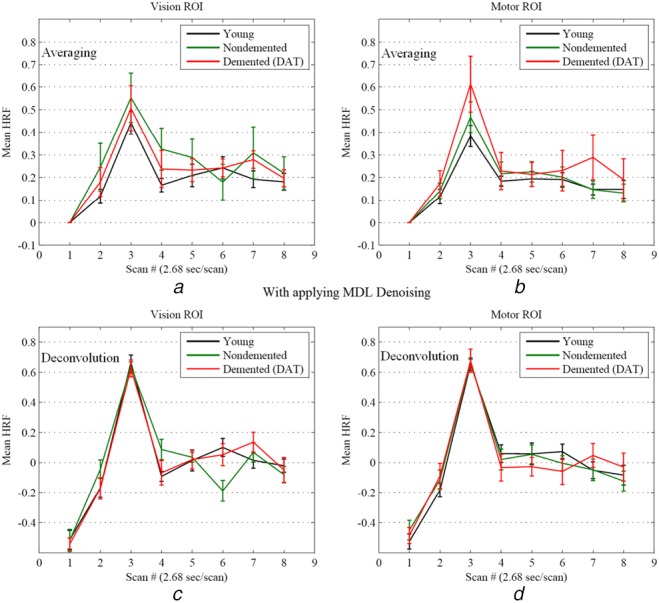
Mean HRF for vision and motor regions extracted from fMRI data with applying MDL denoising *a* Mean HRF due to averaging in vision region (with applying MDL denoising) *b* Mean HRF due to averaging in motor region (with applying MDL denoising) *c* Mean HRF obtained from deconvolution in vision region (with applying MDL denoising) *d* Mean HRF obtained from deconvolution in motor region (with applying MDL denoising). Error bars represent ±1 SEM. The black, green and red solid lines are associated with the mean HRF for the young, non-demented and demented elderly adults, respectively

The effects of noise are here studied on the HRF estimation to compare the HRF extraction methods of averaging (used in earlier works) and deconvolution (this work). Fig. 2 demonstrates the mean HRF extracted after applying MDL denoising fMRI data for visual and motor cortices.

Figs. 2*a*/1*b* and 2*c*/1*d* are associated with the averaging and the deconvolution techniques, respectively. In the case of averaging algorithm, no significant difference at the peak amplitudes is observed between the elderly adults (non-demented and demented) and the young for both motor and vision regions.

Figs. 2*c* and *d* demonstrate the mean HRF extracted by deconvolution after applying MDL denoising for vision and motor ROIs, respectively. A significant difference is not obtained for motor and vision regions either. It may be seen that the results after applying MDL denoising is different with the one found in the earlier subsection for the case of fMRI data without applying MDL denoising. The noise contribution gets larger and more important through age increasing [16, 18, 20, 21]. According to Table 2, it is observed that the percentage of new voxels for the elderly adults (non-demented and demented) is much more than the young. Then, the result of the HRF extraction after applying MDL denoising may be more reliable. Also, it is highlighted that the averaging and deconvolution techniques lead to the same results after applying MDL denoising.

## 4 Declaration of interests

In the step of HRF estimation, deconvolution appears to be much more robust against noise contribution because the related haemodynamic function is, at the same time, optimised through the whole time series scans. Consequently, the averaging method yields contradictory results for HRF peak amplitudes as reported in the previous works (Table 3). Considering deconvolution method, it may be concluded that the HRF peak amplitudes exhibit no significant difference in the visual and motor cortices between the elderly adults (non-demented and demented) and the young. The findings of this study have been summarised in Table 3 in comparison with the previous works. The divergence of earlier works on the results concerning HRF peak amplitude may be easily observed.

**Table 3:** Comparison of HRF peak amplitudes between the elderly adults and the young

Study reference	ROIs	
	Vision	Motor
This work (2017)	[N & D] = [Y]	[N & D] = [Y]
Mohtasib *et al.* (2012)	—	[N] > [Y]
Aizenstein *et al.* (2004)	[N] = [Y]	[N] = [Y]
Huettel *et al.* (2001)	[N] = [Y]	[N] = [Y]
Buckner *et al.* (2000)	[N & D] < [Y]	[N & D] = [Y]
D'Esposito *et al.* (1999)	—	[N] = [Y]
Ross *et al.* (1997)	[N] < [Y]	—
Gauthier *et al.* (2013)	grey matter of the whole brain	
	[N] < [Y]	

[N]: HRF amplitude peak for the non-demented elderly adults.

[D]: HRF amplitude peak for the demented elderly adults.

[Y]: HRF amplitude peak for the young.

=: no significant difference.

## 5 Conclusion

In this paper, a comparative study has been realised on the HRF peaks amplitudes being compared between the young and the elderly adults (non-demented and demented old populations) assuming simple visual and motor tasks. It was shown that the previous works led to conflicting results on the HRF peak amplitudes because of averaging algorithm as well as noise contributions in fMRI data. In fact, fMRI data are heavily noisy particularly in the elderly adults. Accordingly, it is necessary to employ an HRF estimation algorithm which is sufficiently robust against noise. Employing the robust MDL-based wavelet denoising, it was shown that a large per cent of the selected voxels changed in the activation detection at both motor and visual cortices. According to the experimentation results, the area of activated voxels is extended for both the elderly adults and the young. In the step of HRF estimation, deconvolution appears to be much more robust against noise contribution. Considering deconvolution method, it may be concluded that the HRF peak amplitudes exhibit no significant difference in the visual and motor cortices between the elderly adults and the young.
